# The relationship between positive workplace gossip and job satisfaction: The mediating role of job insecurity and organizational identity

**DOI:** 10.3389/fpsyg.2022.989380

**Published:** 2022-11-28

**Authors:** Dawei Wang, Zhaoxiang Niu, Chongyu Sun, Peng Yu, Xiaolong Wang, Qihui Xue, Yixin Hu

**Affiliations:** ^1^Educational Development Research Center of Southern Xinjiang, Kashi University, Kashi, China; ^2^School of Psychology, Central China Normal University, Wuhan, China; ^3^School of Psychology, Shandong Normal University, Jinan, China; ^4^School of Education Science, Kashi University, Kashi, China

**Keywords:** positive workplace gossip, job satisfaction, job insecurity, organizational identity, social information processing theory

## Abstract

From the perspective of social information processing theory and social identity theory, 1,267 employees were selected as the subjects, and the data were statistically analyzed by using Mplus8.0 and SPSS25.0 to explore the relationship between positive workplace gossip and job satisfaction and the role of job insecurity and organizational identity in this relationship. The results showed that there was a significant positive correlation between positive workplace gossip and job satisfaction. Furthermore, job insecurity and organizational identity independently mediated the relationship between positive workplace gossip and employee job satisfaction. In addition, job insecurity and organizational identity played a serial mediating role in the relationship between positive workplace gossip and job satisfaction. The results of the study shed light on how job insecurity and organizational identity were associated with the process of the positive workplace gossip-job satisfaction relationship. Based on the findings, implications and avenues for future research were discussed.

## Introduction

Workplace gossip is common in the work environment; it is one of the channels for employees to communicate in the organization, and plays a very important role in the organization (Irvine and Blessing, 2019). Studies indicated that employees spend approximately 65% of their working time on gossip (Ye et al., 2019), and more than 95% of employees participated in workplace gossip (Grosser et al., 2012), which further demonstrates the prevalence of workplace gossip. Workplace gossip can be divided into positive and negative workplace gossip according to its impact (Ellwardt et al., 2012). The impacts of negative workplace gossip on employees’ work outcomes such as work efficiency, job satisfaction, and work enthusiasm have attracted more research attention (DiFonzo et al., 1994; Blakeley et al., 1996; Baker and Jones, 2006; Yue et al., 2015), but less is known about the impact of positive workplace gossip on employees’ outcomes such as job satisfaction (Chen and Jiang, 2017). Therefore, this study aims to explore the relationship between positive workplace gossip and employees’ job satisfaction.

In addition, previous studies have pointed out that positive workplace gossip can help individuals effectively cope with job insecurity (Jiang et al., 2019) and increase employees’ sense of organizational identity (Ye et al., 2019). Positive workplace gossip may reduce employees’ job insecurity by alleviating their uneasiness at work (Chen and Jiang, 2017; Song et al., 2018; Jiang et al., 2019) and increase employees’ sense of organizational identity (Ye et al., 2019), which, in turn, improve employees’ work efficiency and job satisfaction (Callea et al., 2016; Asif et al., 2019; Di Stefano et al., 2020), suggesting that job insecurity and organizational identity may play a role in explaining the relationship between positive workplace gossip and job satisfaction. But there were no studies to prove that.

According to social information processing theory and social identity theory, positive workplace gossip is an important part of the workplace environment, which includes praising and approving positive information such as work ability, attitude, and performance. When employees encounter positive workplace gossip, such as leaders’ or colleagues’ affirmation of their work ability and performance, they receive and process these positive information, feel recognized by the organization and others, and their demands for respect and self-needs are satisfied, which may reduce their job insecurity, increase their sense of identity with the organization, and ultimately affect their job satisfaction. In other words, positive workplace gossip may indirectly affect employee job satisfaction through job insecurity and organizational approval. This is the second innovation of this study. Thus, this study’s second goal is to examine the mediating effect of organizational identity and job insecurity on the relationship between positive workplace gossip and job satisfaction.

Besides, previous studies have shown that job insecurity is an antecedent variable of organizational identity (Piccoli et al., 2017; Huang, 2019); job insecurity can reduce employees’ organizational identity (Asif et al., 2019; Ali et al., 2020). Thus, job insecurity and organizational recognition of positive workplace gossip may play a serial mediating role in the relationship between job satisfaction and job insecurity. This is the third innovation of this study. Therefore, this study’s third objective is to examine the serial mediating effect of organizational identity and job insecurity on the relationship between positive workplace gossip and job satisfaction.

**Figure 1:**
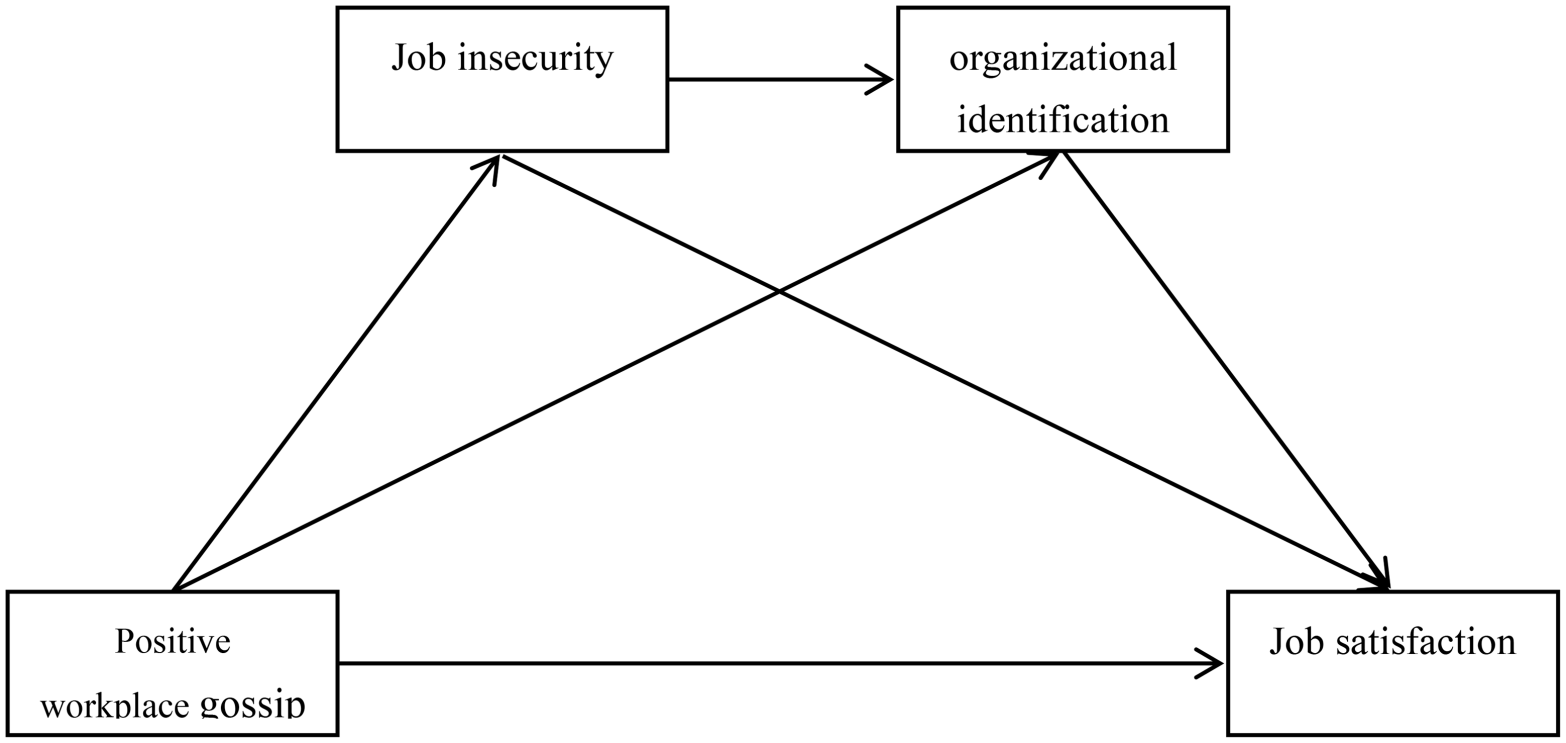
Serial mediating model of positive workplace gossip and job satisfaction.

In summary, based on the theory of social information processing, this study firstly aims to explore the relationship between positive workplace gossip and employee job satisfaction. Second, this study intends to explore the independent mediating role of job insecurity and organizational identity in the relationship between positive workplace gossip and job satisfaction. Finally, this study wants to explore the serial mediating role of job insecurity and organizational identity in the relationship between positive workplace gossip and job satisfaction. The conceptual model diagram is shown in Figure 1.

## Literature review and research hypotheses

### Positive workplace gossip and job satisfaction

Positive workplace gossip refers to the behavior of conveying positive information about an absent third person with a positive personal evaluation between people in appropriate situations (Foster, 2004). Positive workplace gossip can be the recognition and praise of one’s work ability, attitude, and performance. It can be regarded as talking about normative behavior or positive reputation, which may have an impact on the attitude and behavior of the employee being discussed (Brady et al., 2017). Some empirical studies have further proven that positive workplace gossip has a positive impact on employees’ attitudes and behaviors, including improving performance, reducing free-rider behavior and increasing willingness to obey organizational order (Beersma and Van Kleef, 2012; Feinberg et al., 2012; Bai et al., 2019).

However, there are few empirical studies exploring the impact of positive workplace gossip on job satisfaction. Job satisfaction is a kind of reaction of employees to their work situation, which refers to a subjective feeling of whether employees are satisfied with the work environment and the job itself from both psychological and physiological aspects (Hoppock, 1935). According to social information processing theory, people’s attitudes and behaviors are largely influenced by the surrounding social environment. People decide what attitudes and behaviors to adopt through the processing and interpretation of specific social information (Salancik and Pfeffer, 1978). On the one hand, positive workplace gossip is an important part of the workplace environment. The recognition and praise of employees’ work ability, attitude, and performance in the workplace provide employees with a positive working environment and close interpersonal relationships, making employees have a more positive evaluation of the organization and themselves and a higher degree of satisfaction. On the other hand, the prevalence of positive workplace gossip among employees amplifies the positive impact of its related to work, and creates a more positive work atmosphere. Thus, employees will be more satisfied with the subjective response to the work situation, experiencing less pressure performance and feeling higher job satisfaction when they engage in and face positive workplace gossip (Salancik and Pfeffer, 1978). Based on the above analysis, this study believes that positive workplace gossip may improve employees’ job satisfaction. Therefore, the following hypothesis is proposed:

*Hypothesis 1*: Positive workplace gossip will have a positive impact on job satisfaction.

### The mediating role of job insecurity and organizational identity

#### Job insecurity

Job insecurity refers to a sense of helplessness about the sustainability of work in a threatened environment, one of the most important organizational stressors encountered by employees (Greenhalgh and Rosenblatt, 1984; Wang et al., 2020a). Previous studies have shown that job insecurity notably affects individual and organizational outcomes, such as employees’ mental health, job satisfaction, and job performance (Ashford et al., 1989; Sverke et al., 2002). Additionally, job insecurity negatively predicts job satisfaction among workers (Adkins et al., 2001; Chirumbolo and Hellgren, 2003; Feather and Rauter, 2004; Richter and Näswall, 2019; Yeves et al., 2019); that is, employees who feel higher job insecurity experience lower job satisfaction (Hsieh and Huang, 2017; Zhang et al., 2018; Tian et al., 2019; Di Stefano et al., 2020). Therefore, there is a negative correlation between job insecurity and job satisfaction.

In addition, studies have shown that workplace gossip is one of the factors affecting individual job insecurity; positive workplace gossip can enable individuals to effectively deal with job insecurity (Jiang et al., 2019). Employees buffer the negative impact of job insecurity on workplace friendships by participating in positive workplace gossip and pointing out negative workplace gossip to others (Jiang et al., 2019). According to social information processing theory, after receiving positive gossip in the workplace (such as positive comments or praise from supervisors or colleagues), employees have more positive subjective feelings about themselves and their work, experiencing a higher sense of self-efficacy and control over their work. When faced with job insecurity, employees can effectively deal with job insecurity (Jiang et al., 2019) and then experience higher job satisfaction (Richter and Näswall, 2019). On the other hand, employees describe themselves as “good employees” by actively participating in positive workplace gossip so that they can meet the values and expectations of the organization, thus reducing their job insecurity and experiencing higher job satisfaction (Jiang et al., 2019; Di Stefano et al., 2020). In summary, gossip is a predictor of job insecurity, and job insecurity played a buffering role in the relationship between positive workplace gossip and job satisfaction based on cognition appraisal theory. This study proposes the following hypothesis:

*H2*: Job insecurity mediates the relationship between positive workplace gossip and job satisfaction.

#### Organizational identity

Organizational identity means that when members more strongly identify with the organization and its values, the organization will become an important part of its members’ live, just as members are an important part of the organization (Cheney and Tompkins, 1987). In addition, existing studies have pointed out that negative workplace gossip can reduce organizational identity and service performance of hotel employees (Ye et al., 2019). Negative workplace gossip threatens the four basic needs of victimized employees, reduces their sense of identity with the organization, decreases their interest in improving the effectiveness of the organization, and declines their investment in prescribed customer service and customer-oriented organizational citizenship behavior (CO-OCB), thus reducing their job satisfaction (Ellemers et al., 2004; Lu et al., 2016). In the same way, according to social identity theory, in most social situations, individuals tend to regard themselves as typical members of groups such as organizations (Ashforth and Mael, 1989). They identify strongly with a particular group when it satisfies their four basic needs, including positive self-esteem, belonging, control, and the need for a good life. When the basic needs are met, their identity with the group will improve (Ashforth and Mael, 1989; Hogg and Terry, 2000; Smidts et al., 2001). As a pleasant experience, positive workplace gossip can meet the basic needs of employees and enhance their organizational identity. First, the positive comments and evaluations of positive workplace gossip make employees feel that they are respected and appreciated by their supervisors and colleagues, thus making positive judgments about themselves. Second, positive workplace gossip sends a signal to employees that they are accepted by the organization, increasing their sense of belonging. Third, positive workplace gossip increases employees’ sense of control because employees can obtain expected information and evaluation from supervisors and colleagues. Finally, positive workplace gossip hints at employees’ sense of value and increases their sense of meaning (Ashforth and Mael, 1989; Ye et al., 2019). Therefore, positive workplace gossip increases employees’ sense of organizational identity.

Importantly, previous studies have shown that organizational identity plays an important role in employees’ job satisfaction (e.g., Brown, 1969; Schneider, 1972; Rotondi, 1975; O’Reilly and Chatman, 1986). People with a high sense of organizational identity will view their actual work situation more positively, leading to higher job satisfaction (Van Dick et al., 2004; Tang, 2009; Wang et al., 2019a,b,c; Jeanson and Michinov, 2020). Meanwhile, individuals with a high organizational identity are more willing to work for the company and derive more happiness from work (Hwang and Jang, 2020; Mete et al., 2016).

In summary, positive workplace gossip and organizational identity may affect employees’ job satisfaction, and positive workplace gossip may affect employees’ organizational identity (Gerstner and Day, 1997; Van Dick et al., 2004; Riketta, 2005; Riketta and Van Dick, 2005; Dulebohn et al., 2012; Tan et al., 2020). Consistent with the theory of social identity, when employees experience higher positive workplace gossip, the higher their organizational identity regarding work will be and the higher their job satisfaction will be (Ashforth and Mael, 1989). Therefore, employees’ organizational identities may play a mediating role in the relationship between positive workplace gossip and employee job satisfaction. It is assumed that:

*H3*: Organizational identity plays a mediating role in the relationship between positive workplace gossip and job satisfaction.

### The serial mediating role of job insecurity and organizational identity

Job insecurity and organizational identity may play an independent mediating role in the relationship between positive workplace gossip and job satisfaction, but the existence of a serial mediator remains to be further studied. Studies have explored the relationship between job insecurity and organizational identity, pointing out that job insecurity is an antecedent variable of organizational identity (Piccoli et al., 2017; Huang, 2019). The higher the employees’ job insecurity, the lower their organizational identity is likely to be (Callea et al., 2016; Song et al., 2018; Asif et al., 2019; Kim, 2019). Negative factors in an organization (such as job insecurity) will affect the construction of employees’ organizational identity, break the bond between employees and the organization and thus lead to the decline of employees’ job satisfaction (Ashforth and Mael, 1989). According to social information processing theory, we believe that positive workplace gossip provides a positive workplace environment. In a positive workplace environment, positive workplace gossip makes employees more likely to experience recognition from the organization and colleagues. It makes them have a more positive evaluation of the organization and themselves, which may reduce their job insecurity. The reduction of job insecurity increase employees’ confident about keeping their jobs and further satisfies employees’ basic needs, such as self-efficacy and sense of control, which leads to a higher sense of identity with the organization and ultimately improves their job satisfaction (Ashforth and Mael, 1989). Therefore, we speculate that positive workplace gossip may improve employees’ organizational identity and ultimately improve their job satisfaction by reducing their sense of job insecurity. In other words, we hypothesize that job insecurity and organizational identification of positive workplace gossip and job satisfaction serve as serial mediators. In summary, this study proposes the following hypothesis:

*H4*: Job insecurity and organizational identification play a serial mediating role in the relationship between positive workplace gossip and job satisfaction.

## Materials and methods

### Participants and procedures

A random sampling method was adopted to select 1,267 employees in a state-owned enterprise in China as the research objects, among whom 789 were male (62.3%), and 478 were female (37.7%). The average age was 37.86 years old (*SD* = 7.16). The marital status of the study subjects was as follows: married (87.6%), unmarried (8.1%), and others (4.3%). The subjects’ educational levels were: 1.0% with junior middle school education, 27.6% with a high school or technical secondary school education, 30.6% with a junior college education, 35.8% with undergraduate education, and 4.9% with a master’s degree.

The human subjects involved in this study all conformed to the Academic Committee of Shandong Normal University’ s ethical standards, as well as the 1964 Helsinki Declaration and its subsequent revisions. Participants volunteered to participate in the study and were paid to do so. Participants provided informed consent and verbal consent to participate before completing the questionnaire. A brief guide describes the purpose of the investigation and data confidentiality procedures. It took participants approximately 15 min to complete all the questionnaires. Analyses were conducted using SPSS 25.0. Mediation analyses made use of Mplus 8.0, and we used 0.05 as the critical values for our test of hypothesis depending upon the test statistic.

### Measurement tools

#### Positive workplace gossip scale

The positive workplace gossip scale compiled by Brady et al. (2017) and revised by Xuan and Majid (2018) was adopted to measure employees’ positive workplace gossip. The scale includes 10 questions on two dimensions, including positive workplace gossip about co-workers and positive workplace gossip about the boss. Examples include questions such as “Complimented on your boss’s actions during a conversation with a colleague.” A 7-point Likert scale was used, and the higher the score, the higher the positive workplace gossip. The Cronbach’s alpha for this scale was 0.979.

#### Job satisfaction scale

The job satisfaction scale compiled by Hackman and Oldham (1980) and revised by Shu and Liang (2015) was adopted to measure employees’ job satisfaction. The scale contains three questions, with sample questions such as “I am generally satisfied with the work I have done in this position.” A 5-point Likert scale was used, and the higher the score was, the higher the job satisfaction. The Cronbach’s alpha for this scale was 0.948.

#### Job insecurity scale

The work insecurity scale compiled by Borg and Elizur (1992) and revised by Zhang et al. (2014) was used to measure employees’ job insecurity. The scale includes two dimensions of cognitive job insecurity and emotional job insecurity. There are 7 questions in total. Cognitive job insecurity is scored in reverse on a scale of 1–4, and emotional job insecurity is scored positively on a scale of 5–7. Example questions include “I think my job is secure.” A 7-point Likert scale was used, and the higher the score was, the higher the job insecurity. The Cronbach’s alpha for this scale was 0.779.

#### Organizational identity scale

The organizational identity scale compiled by Smidts et al. (2001) and revised by Gao and Zhao (2014) was used to measure employees’ organizational identity. This scale contains 5 questions, with example questions such as “I have a strong sense of belonging to our organization.” A 5-point Likert scale was used, and the higher the score was, the stronger the organizational identity. The Cronbach’s alpha for this scale was 0.970.

## Results

### Common method bias

This study adopted the Harman single-factor technique to estimate the influence of common method bias. The results showed that there were 4 factors emerged, with an interpretation rate of the population variance of 86.75%. The interpretation rate of the first common factor was 38.44%, indicating that there was no serious common method bias in this study (Podsakoff et al., 2003).

### Correlation analysis

Table 1 shows that positive workplace gossip was positively correlated with job satisfaction and organizational identity (*r* = 0.338, *p* < 0.01; *r* = 0.327, *p* < 0.01), while positive workplace gossip was negatively correlated with job insecurity and age (*r* = −0.136, *p* < 0.01; *r* = −0.118, *p* < 0.01). Additionally, job satisfaction was negatively correlated with job insecurity (*r* = −0.181, *p* < 0.01). There was a positive correlation between job satisfaction and organizational identity (*r* = 0.711, *p* < 0.0 l) and a negative correlation between job insecurity and organizational identity (*r* = 0.182, *p* < 0.0 l).

**Table 1:** Describes statistics.

Variables	*M*	*SD*	1	2	3	4	5	6	7	8	9
Positive workplace gossip	4.01	1.80	−								
Job insecurity	3.36	1.13	−0.136^**^	−							
Organizational identity	4.26	0.90	0.327^**^	−0.182^**^	−						
Job satisfaction	4.20	0.87	0.338^**^	−0.181^**^	0.711^**^	−					
Gender	1.38	0.49	−0.102^**^	−0.020	0.024	0.058^*^	−				
Marital status	1.17	0.48	0.019	−0.010	0.010	0.000	0.093^**^	−			
Years	37.86	7.16	−0.118^**^	0.038	0.020	0.038	−0.221^**^	−0.129^**^	−		
Work time	15.81	8.27	−0.088^**^	0.051	0.019	0.033	−0.286^**^	−0.109^**^	0.916^**^	−	
Education	3.16	0.92	0.093^**^	−0.240^**^	−0.003	−0.022	0.102^**^	0.000	−0.240^**^	−0.315^**^	-

*N* = 1,267.

* *p* < 0.05;

** *p* < 0.01;

** *p* < 0.001.

Independent sample *t*-test results showed that there was a significant difference between male and female job satisfaction (*t* = −2.071, *p* = 0.039). Under different education levels, there was no significant difference in job satisfaction (*F* = 0.264, *p* = 0.901). There was no significant difference in job satisfaction among employees of different ages (*F* = 1.203, *p* = 0.201). There was no significant difference in job satisfaction between married and unmarried employees (*t* = 0.522, *p* = 0.593).

### The mediating role of job insecurity and organizational identity in the relationship between positive workplace gossip and job satisfaction

**Figure 2:**
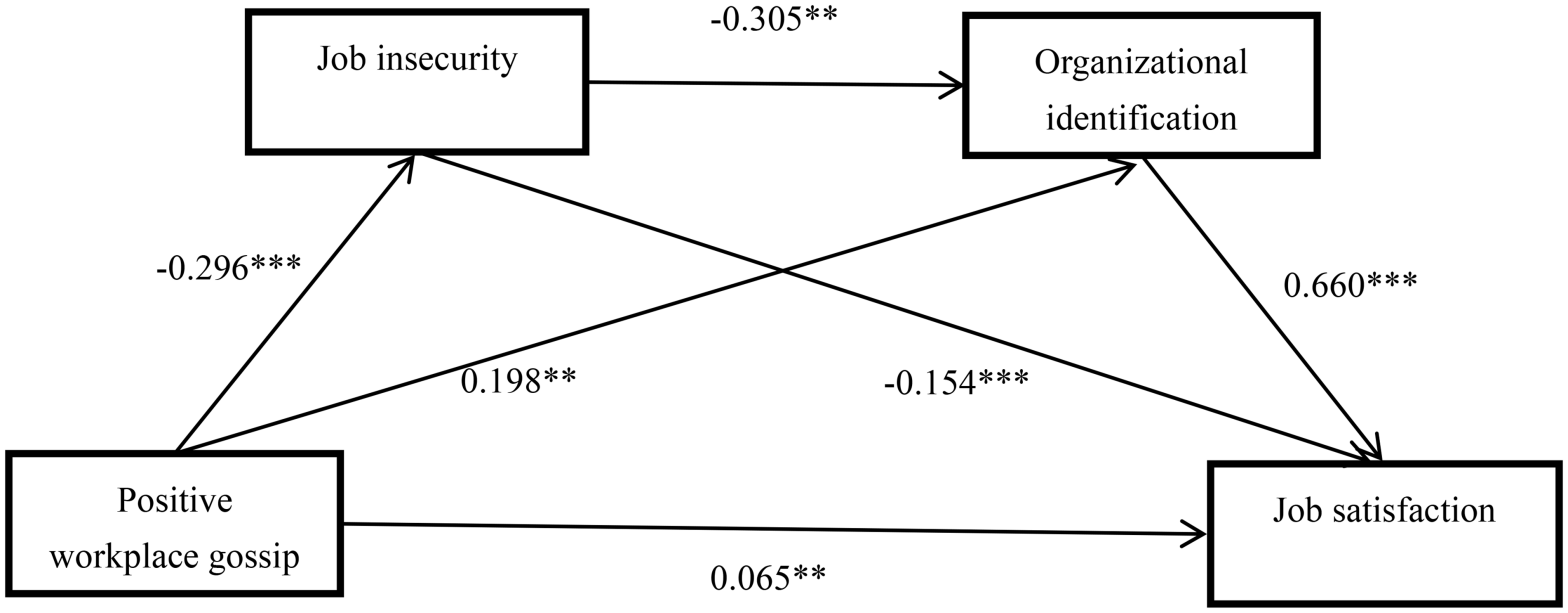
Serial mediating model of positive workplace gossip and job satisfaction. **p < 0.01; ***p < 0.001.

The Mplus program was used to test the hypothetical model, specifying 95% confidence intervals and 5,000 bootstrap resamples (Hayes, 2013; Wen and Ye, 2014). Because demographic variables were not associated with job satisfaction, they were not included in the analysis. Tables 2–4 and Figure 2 show the results of the structural equation model analysis, supposing χ^2^ = 660.879, *df* = 245, *p* < 0.01, χ^2^/*df =* 2.697*, CFI* = 0.992, *TLI* = 0.990, *RMSEA* = 0.037 (90% *CI =* [0.033, 0.040]), *SRMR* = 0.059), and good fitting of the model data (Wen et al., 2003; Hayes, 2013).

**Table 2:** Results of confirmatory factor analysis of the measurement models.

Measurement models	*χ*2	*df*	*RMSEA*	*CFI*	*TLI*	*SRMR*
Four-factor (A, B, C, D)	660.879	246	0.992	0.992	0.990	0.036
Three-factor (A,B + C, D)	5296.837	249	0.126	0.901	0.881	0.124
Two-factor (A+ B + C, D)	12492.924	251	0.196	0.761	0.714	0.241
One-factor (A+ B + C + D)	14494.379	252	0.211	0.721	0.668	0.240

Abbreviations: A, positive workplace gossip; B, job insecurity; C, organizational identity; D, job satisfaction; RMSEA, root-mean-square error of approximation; SRMR, standardized root-mean-square residual; CFI, comparative fit index; TLI, Tucker-Lewis index.

**Table 3:** The mediating role of job insecurity and organizational identity.

	Outcome: Job insecurity			Outcome: Organization identification			Outcome: Job satisfaction		
	*b*	*SE*	*t*	*b*	*SE*	*t*	*b*	*SE*	*t*
Gender							0.056	0.02	2.836^**^
Marital status							−0.017	0.013	−1.276
Years							0.061	0.027	2.253^*^
Work time							−0.011	0.040	−0.283
Education							−0.031	0.020	−1.552
positive workplace gossip	−0.296	0.025	−11.765^***^	0.198	0.023	8.711^***^	0.065	0.025	2.633^**^
job insecurity				−0.305	0.049	−6.290^***^	0.660	0.034	19.163^***^
organizational identity							−0.154	0.038	−4.043^***^
R^2^	0.088			0.168			0.574		
F	5.705^***^			4.628^***^			18.147^***^		

* *p* < 0.05;

** *p* < 0.01;

*** *p* < 0.001.

**Table 4:** Test of mediation of job insecurity and organizational identity on the relationship between positive workplace gossip and job satisfaction: Bootstrap results.

	Estimate	*SE*	*t*	*p*	BootLLCI	BootULCI
Total Ind: JI → JS	0.128	0.014	9.249	0.000	0.105	0.152
Indirect effect *via* JI	0.025	0.008	3.170	0.002	0.018	0.042
Indirect effect *via* OD	0.071	0.009	8.250	0.000	0.059	0.092
Indirect effect *via* JI and OD	0.032	0.007	4.917	0.000	0.02	0.043
Total	0.164	0.017	9.858	0.000	0.134	0.184

JI = job insecurity, OD = organizational identity, JS = job satisfaction.

The study tested each path of the hypothetical model and found that positive workplace gossip significantly predicted job satisfaction, as shown in Figure 2 and Table 3 (*β* = 0.296, *SE* = 0.025, *p* < 0.001); therefore, Hypothesis 1 was true. The indirect effect of workplace gossip on job satisfaction through job insecurity was significant, as shown in Table 4 (*β* = 0.025, *SE* = 0.008, *p* < 0.01); therefore, Hypothesis 2 was true. The indirect effect of workplace gossip on job satisfaction through organizational identification was significant, as shown in Table 4 (*β* = 0.071, *SE* = 0.009, *p* < 0.001), therefore Hypothesis 3 was true. Positive workplace gossip had a significant indirect effect on job satisfaction through a serial of job insecurity and organizational identification, as shown in Table 4 (*β* = 0.032, *SE* = 0.007, *p* < 0.001); therefore, Hypothesis 4 was true.

## Discussion

### Theoretical applications

In response to the call of current positive psychology, this paper discussed workplace gossip from a positive perspective, which was beneficial to change the past focus on the negative aspects of workplace gossip. This paper introduced two variables affecting job satisfaction, job insecurity, and organizational identity; constructed a serial mediation model of the relationship between positive workplace gossip and job satisfaction; discussed the mechanism of positive gossip driving job satisfaction; and tested the mediating effect of job insecurity and organizational identity.

First, this study verified the significant positive relationship between positive workplace gossip and job satisfaction, supporting Hypothesis 1. While the positive impact of positive workplace gossip was gaining traction, the relationship between positive workplace gossip and job satisfaction had not been studied. According to social information processing theory, positive workplace gossip is an important part of the workplace environment, which includes praising and approving positive information such as work ability, attitude, and performance. Individuals interpret this positive information related to themselves and experience recognition from organizations and colleagues, which includes more positive feelings, less pressure performance, and higher job satisfaction (Di Stefano et al., 2020). In addition, this study enriched the literature on the role of positive workplace gossip because most of the previous studies focused on the impact of negative workplace gossip (Bosson et al., 2006; Ellwardt et al., 2012; Yeves et al., 2019) but ignored the impact of positive workplace gossip (Wu et al., 2018b; Ye et al., 2019). The results of this study not only reflected the expectations of previous studies on workplace gossip (Foster, 2004; Beersma and Van Kleef, 2012; Sun and Mai, 2016; Wu et al., 2018a,b; Tian et al., 2019; Ye et al., 2019) but also added relevant theoretical literature on positive workplace gossip, providing empirical evidence of positive workplace gossip on employees’ job satisfaction.

Our study also found significant gender differences in employee job satisfaction, the result that was consistent with previous studies (Magee, 2015; Yang and Jeong, 2020). Specifically, men (*M* = 4.16) had lower job satisfaction than women (*M* = 4.26). Research have shown that men are more focused on material or external rewards than women, which may explain why many studies have found that men report lower job satisfaction than women (Magee, 2015). Because this leads to men putting “golden handcuffs” on themselves, in the sense that they feel more strongly compelled than women to continue to do work that they have no intrinsic motivation to do, and they are not particularly proud of it (Rynes et al., 2004; Magee, 2015).

Second, this study examined the mediating role of job insecurity in the relationship between positive workplace gossip and job satisfaction, which supported Hypothesis 2. The empirical results of this study confirmed the hypothesis of previous studies; namely, positive workplace gossip could reduce employees’ job insecurity (Jiang et al., 2019), and the reduction of job insecurity could increase employees’ job satisfaction (Jiang et al., 2019; Richter and Näswall, 2019). The results further indicated that job insecurity was an important transmission mechanism between positive workplace gossip and job satisfaction and that employees’ perception of the workplace environment affected employees’ perception of their jobs. According to social information processing theory, positive workplace gossip creates a positive work environment for our employees. This positive work environment reduces employees’ job insecurity, increases positive organizational aspects of the attention of employees, and leads to more positive organizational evaluation so that employees perceive higher job satisfaction (Salancik and Pfeffer, 1978; Tian et al., 2019; Yeves et al., 2019; Di Stefano et al., 2020). The model of job insecurity in this study helps explain how positive workplace gossip influences employees’ job satisfaction.

Third, this study found the mediating role of organizational identity in the relationship between positive workplace gossip and job satisfaction, which supported Hypothesis 3. The results of this study provided evidence for previous research hypotheses that organizational identity (Ye et al., 2019) and the improvement of organizational identity could increase employees’ job satisfaction (Van Dick et al., 2004; Mete et al., 2016). The results further showed that positive workplace gossip might increase employees’ job satisfaction by increasing their organizational identity, that is, the organizational identity part mediated the relationship between positive workplace gossip and employees’ job satisfaction. According to social identity theory, we believe that organizational identification is one of the positive workplace gossip effect outcomes because positive workplace gossip contains positive comments and evaluation, which can satisfy employees’ positive self-esteem, sense of belonging, sense of control, and demand for the good life, meeting employees’ basic needs. Correspondingly, employees’ organizational identity increases and they experience higher job satisfaction (Ashforth and Mael, 1989; Lu et al., 2016; Ye et al., 2019; Tan et al., 2020). This organizational identity model helps to explain the mechanism by which positive workplace gossip affects employee job satisfaction.

Finally, the results also examined the serial mediating effect of job insecurity and organizational identity, suggesting that job insecurity and organizational identity played a serial mediating role in the relationship between positive workplace gossip and job satisfaction, supporting Hypothesis 4. The research results provided a new theoretical framework to explain the mechanism of positive workplace gossip on employees’ job satisfaction; that was, positive workplace gossip might reduce employees’ job insecurity, increased employees’ organizational identity and ultimately improved employees’ job satisfaction (Mete et al., 2016; Chen and Cai, 2017; Huang, 2019; Tian et al., 2019; Yeves et al., 2019). Consistent with social information processing theory, we believe that positive workplace gossip includes positive evaluations and comments that shape a positive work environment, make employees positively evaluate the organization and themselves, and cause employees to experience a greater sense of security and self-efficacy in the organization, thus reducing their job insecurity (Salancik and Pfeffer, 1978; Chen and Cai, 2017). Then, according to social identity theory, we believe that employees’ job insecurity is reduced, which further meets their basic needs, improves their sense of identity with the organization, and improves their job satisfaction (Ashforth and Mael, 1989; Mete et al., 2016; Yeves et al., 2019).

### Implications for practical applications

First, the study results suggested that employees were more satisfied with their jobs when they received more positive workplace gossip. Therefore, business management organizations can implement effective strategies to play the role of positive workplace gossip. First, given the positive impact of positive workplace gossip, organizations should disseminate positive workplace gossip and create an environment for disseminating positive workplace gossip (Wu et al., 2018a,b). Second, the organization can organize activities and relevant knowledge training about workplace gossip to increase employees’ understanding of workplace gossip and the better use of it. Third, in an ambiguous environment, workplace gossip is especially common (Difonzo and Bordia, 2007). Therefore, to better exert the positive influence of workplace gossip, organizations should also establish convenient and efficient workplace communication channels.

Second, in addition to determining the impact of positive workplace gossip on job satisfaction, this study also found that employees’ job insecurity and organizational identity were important mediators linking positive workplace gossip with job satisfaction. Therefore, the organization can reduce employees’ job insecurity, increase employees’ organizational identity and increase the positive influence of positive workplace gossip to improve employees’ job satisfaction. For example, by improving employees’ organizational identity and increasing the positive impact of workplace gossip on employees’ job satisfaction, organizations can evaluate the sense of personal self-efficacy and the fit of personal values and organizational values when recruiting new employees (Fuller et al., 2006). Organizations can regularly provide employees with relevant stress relief training and organizational culture team building (Fuller et al., 2006). The organization can also disseminate positive information about the organization through internal communication tools (such as email communication) and release training information about employees that can improve their abilities to promote employees’ identification with the organization (Tian et al., 2019; Wang et al., 2020b), increasing employees’ job satisfaction. Organizations can also clarify organizational goals for employees or authorize them to participate in decision-making to enhance their understanding of organizational goals and increase organizational identity (Tian et al., 2019; Wang et al., 2020a) increasing the job satisfaction of employees.

In addition, to reduce the negative impact of job insecurity on job satisfaction, organizations can reduce job insecurity by improving employees’ employment predictability and controllability. Although economic forces beyond employers’ control may determine job insecurity, organizations can improve employees’ perceptions of employment control and predictability by promoting open and clear communication about organizational change (De Witte, 2005; Wang et al., 2019, 2020a). The organization can also encourage employees to participate in the decision-making process and ensure that fair procedures are always in place to help employees feel safe (De Spiegelaere et al., 2014; Probst et al., 2018). Organizations can also take intervention measures or training programs, such as attribution training programs, to reduce employees’ job insecurity and further increase the positive impact of workplace gossip on employees (Jiang et al., 2019; Wang et al., 2019), increasing the positive impact of positive workplace gossip in the workplace on employees’ job satisfaction.

### Limitations and future research trends

This study was not without its limitations. First, there was concern about common methodology bias (CMV; Podsakoff et al., 2003) because all the assumptions in this study regarding job insecurity, organizational identity, and job satisfaction in terms of positive workplace gossip were tested using self-reported questionnaires. Although the questionnaire design of multiple time points and sources was adopted in this study, it was still a cross-sectional study in nature, which can only explore the internal mechanism and cannot deduce the causal relationship between research variables. Therefore, future studies can consider the cross-lagged panel design to determine the cause-and-effect relationship between positive workplace gossip, job insecurity, organizational identity, and job satisfaction and to test the robustness of the conclusions of this study.

Second, the study’s generality may be limited because the sample data were collected from one Chinese company. Employees may be exposed to different types of workplace environments at the same time, such as leadership style (Tu et al., 2017; Boamah et al., 2018; Wang et al., 2020c), organizational support (Griffin et al., 2001; Abou Hashish, 2017), and working atmosphere (Abou Hashish, 2017). When considering other types of organizational environments, it is worth further exploring whether positive workplace gossip has the same impact on employees’ job insecurity, organizational identity, and job satisfaction. Future research could control for other types of work environments to test the unique impact of positive workplace gossip beyond these underlying factors. This will help people better understand the impact of positive workplace gossip. In addition, the results may vary according to different cultures and types of enterprises. Future research could also replicate the study using Western samples or controlling for cultural characteristics.

Third, this study founds that positive workplace gossip could reduce employees’ job insecurity, increase their organizational identity, and ultimately increase their job satisfaction at the individual level. Given the importance of workplace gossip on organizations, future research could explore the impact of workplace gossip at the team level. For example, it would be interesting to study whether team-level service atmosphere, service performance, and service-oriented creativity are affected by positive workplace gossip (Zhou et al., 2020; Zou et al., 2020). Such research will also help us better understand the impact of positive workplace gossip on organizations.

Finally, the results suggested that job insecurity and organizational identity only partially explained the relationship between positive workplace gossip and job satisfaction, suggesting that other mediating factors may exist. Therefore, future research is necessary to explore the influence of more mediating variables on positive workplace gossip, such as work motivation, organizational commitment, organizational fairness, emotional exhaustion, psychological capital, and job investment (De Spiegelaere et al., 2014; Aybas et al., 2015; Wu et al., 2018a; Wang et al., 2020a).

## Conclusion

Positive workplace gossip positively predicted employees’ job satisfaction. Positive workplace gossip helped increase employees’ job satisfaction. Job insecurity and organizational identity played a serial mediating role in the relationship between positive workplace gossip and job satisfaction. In other words, positive workplace gossip could improve employees’ job satisfaction by reducing their job insecurity and increasing their organizational identity.

## Data availability statement

The original contributions presented in the study are included in the article/supplementary material, further inquiries can be directed to the corresponding authors.

## Ethics statement

The studies involving human participants were reviewed and approved by the Academic Committee of Shandong Normal University’s ethical standards. The patients/participants provided their written informed consent to participate in this study.

## Author contributions

DW: model building, writing. ZN: writing and revisions. CS: data analysis. PY, XHQ: data collection and analysis, revisions. XLW: revisions. YH: model building, revisions, supervision. All authors contributed to the article and approved the submitted version.

## Conflict of interest

The authors declare that the research was conducted in the absence of any commercial or financial relationships that could be construed as a potential conflict of interest.

## Publisher’s note

All claims expressed in this article are solely those of the authors and do not necessarily represent those of their affiliated organizations, or those of the publisher, the editors and the reviewers. Any product that may be evaluated in this article, or claim that may be made by its manufacturer, is not guaranteed or endorsed by the publisher.
